# Atlas of Immune Cell Populations of the Inflamed Mammalian CNS

**DOI:** 10.3390/cells7050039

**Published:** 2018-05-08

**Authors:** Alex Kalyuzhny

**Affiliations:** Department of Neuroscience, UMN Twin Cities, 6-145 Jackson Hall, 321 Church St SE, Minneapolis, MN 55455, USA; kalyu001@umn.edu

Two processes are known to take place during neuroinflammation: (i) resident immune cells are activated and (ii) inflammatory leukocytes in the periphery begin to infiltrate the central nervous system (CNS). As a result of the combination of bona fide neuronal immune system cells with infiltrating leukocytes from the periphery, it is difficult to characterize these diverse populations of cells and to understand their function with regard to age-related inflammation and neurodegeneration. At present, the only technique that allows the characterization of immune system cells is immunohistochemistry (IHC), which relies on the detection of different immune cell biomarkers in neuronal tissue sections. A serious drawback of IHC is its inability to discriminate between blood-derived infiltrating leukocytes and resident neuronal immune cells. 

To tackle this challenging task, a group of twelve scientists from three research centers including the University of Zurich (Switzerland), Charité—Universitätsmedizin in Berlin (Germany), and the University of Virginia (USA) utilized high parametric mass cytometry, IHC, 22-color fluorescence cytometry, as well as reporter and fate-mapping systems [1]. Through a combination of these techniques, they (i) created a high-dimensional, single-cell proteome atlas of immune cell populations, (ii) discovered the subsets of CNS-resident phagocyte populations, and (iii) even defined entire immune cell landscapes during aging, neurodegeneration, and neuroinflammation. For example, using high-dimensional cytometry in combination with neural network-based algorithms, the authors identified separate populations of microglia, CNS border-associated macrophages (BAMs), bone marrow-derived dendritic cells (DCs), as well as monocytes, and corroborated their findings with functional, genetic, and fate-mapping approaches. They also identified multiple subsets of DCs within the unaffected CNS of mice. One of their most interesting findings was the identification of specific subsets of reactive microglia associated with aging. The authors questioned a previous conclusion which posited that CD44 alone can be a reliable biomarker of CNS-infiltrating cells [2]. In this study, they found that CD44 is also expressed by steady-state CNS-resident leukocytes and can be upregulated in these cells by microglia and BAMs during experimental autoimmune encephalomyelitis (EAE). Through an analysis of the differential expression of biomarkers such as CD38, Lyve-1, MHCII, and CCR2, the researchers reported four previously uncharacterized BAM subsets. They found that, at the peak of EAE disease, BAMs lose their heterogeneity and almost exclusively co-express CD38 and MHCII.

Notably, this group of researchers used sophisticated, state-of-the-art techniques. For instance, they designed a 43 heavy-metal, isotope-tagged surface antibody panel for mass cytometry complemented it with 22-color fluorescence cytometry to identify all the major leukocytes in the CNS; they then used IHC to locate specific cell populations within the CNS. Mass cytometry allows for a more thorough examination of cell function as it can simultaneously measure more than 40 parameters for millions of cells. At the single-cell level it provides a high resolution of the functional and phenotypic complexity of biological systems which is impossible to obtain by fluorescent cytometry alone. 

The results of this study impact our understanding of the role of immune system cells in CNS pathology, including aging, neurodegeneration, and neuroinflammation. The results of this study have also laid the foundation for creating a high-dimensional, single-cell proteome atlas of immune cell populations of the mammalian CNS, which can be used to study individual leukocyte populations and clearly demarcate their roles in CNS pathologies. 
